# Food Innovation as a Means of Developing Healthier and More Sustainable Foods

**DOI:** 10.3390/foods10092069

**Published:** 2021-09-01

**Authors:** Adrián Rabadán, Roberto Nieto, Rodolfo Bernabéu

**Affiliations:** Higher Technical School of Agriculture and Forestry Engineering, University of Castilla-La Mancha, 02071 Albacete, Spain; Roberto.nieto@alu.uclm.es (R.N.); Rodolfo.Bernabeu@uclm.es (R.B.)

**Keywords:** novel foods, functional food, food by-product, sustainability, food neophobia

## Abstract

The current demand for healthy and sustainable foods has encouraged the development of new alternatives even in traditional products. Improved foods may be produced by reducing the amount of some ingredients, adding new ones, or replacing traditionally used ingredients for others. By reformulating their products, manufacturers can offer healthier choices for an ever-growing number of consumers interested in maintaining a balanced diet. In addition, the market demand for more sustainable foods contributes to a lower environmental impact in their production. In this regard, current areas of interest include the production of foods using a lower number of inputs, as well as the utilization of food by-products, to improve the amount and quality of available foods. Another aspect to be considered is that not all consumers are willing to eat foods produced with new ingredients or novel technologies. Hence, the development of innovations in food products should take into account the influence of so-called “consumer food neophobia”.

Consumers are increasingly aware of the composition of their food and the way in which it is produced. These new demands have boosted food innovation. Within food innovation studies, two main trends have dominated the studies developed in Western countries in recent years [1]. The first is the connection between food and health, which has encouraged the production of health-promoting foods. The second is the study of the impact of food production on the environment and the identification of new strategies (e.g., meat replacement) in order to reduce this impact.

Although an increasing number of consumers are open to trying novel foods [2], food innovation may face rejection from consumers that demand more natural food or foods produced using traditional production schemes [3]. This is referred to as consumer neophobia, which has been defined as the reluctance of consumers to try new or unfamiliar foods. This expected rejection of novel foods is one of the main issues that the implementation of food innovation strategies will have to face in the near future.

The current trend of innovations designed to develop more healthy and sustainable foods includes a wide range of very different approaches. Two approaches that have received major attention are the substitution of food components and the development of new production technologies. In this regard, new starches and other hydrocolloids have expanded the use and substitution of many ingredients [4], and traditional cereal flours have been replaced by other flours such as pulse flours [5] or nut flours [6,7]. New production methods include, for example, the use of critical CO_2_ to conduct the extraction of different components [8] and the use of ultrasound assisted extraction or microwave assisted extraction [9,10]. From the point of view of consumers, studies have focused on consumers’ attitudes towards organic products [11], gluten-free products [6], and the various kinds of innovations that result in the production of more natural or more sustainable foods [3,12].

This Special Issue provides a multidisciplinary view of food innovation including studies focused on the production of healthier and more sustainable foods by using novel ingredients, food by-products, or new food production processes. Additionally, studies about consumers’ perceptions of food innovation or innovative foods have also been considered.

Rábago-Panduro et al. [13] investigated the effect of pulsed electric fields on the extraction yield and stability of oils obtained from pecan nuts (*Carya illinoinensis* (Wangenh. K. Koch)). In their study, the authors evaluated how three specific energy inputs affected oil extraction yield, oil stability, the microstructure of kernels and their by-products’ phenolic compounds and antioxidant capacity. The results show that the use of pulsed electric fields does not improve oil extraction yield, showing that factors such as the moisture or microstructure of kernels play a key role in their effectiveness.

Gómez-Saéz et al. [14] evaluated the effect of three concentrations of saffron on vacuum-packed dry-cured ham. In this study, the authors evaluated several factors, including pH, color, sensorial quality and the safranal content of ham, at 0, 7, 14, 28, and 60 days of storage. Their results show that the addition of saffron did not affect the pH or the color of ham stored for 28 days. However, the storage period affected pH values with a decline observed from day 28. Regarding sensory analysis, significant differences were found in visual appearance, flavor, and odor at different days of storage. The safranal content also showed variations with the time at days 14 and 60.

Rabadán [15] studied consumers’ attitudes towards wine innovation using a sample of 400 Spanish wine consumers using several scales, including the “Wine Neophobia Scale”. His results show that four different segments of consumers exist, showing different attitudes towards technological innovation in the wine sector. The group that shows more positive attitudes towards technological innovation (product and process innovation) is formed by consumers with the highest incomes and levels of education. With that in mind, he concludes that innovations in the wine sector should be directed to consumers within that segment.

In order to improve the nutritional and sensory characteristics of cookies, Martínez et al. [6] studied the effect of the substitution of wheat flour by seed defatted flours in cookies’ elaboration. Four different defatted flours were used: flax, sesame, chia, and poppy. The results show that the use of defatted seed flours resulted in cookies with a higher protein content and a lower content of carbohydrates. Regarding sensory analysis, sesame and flax cookies obtained similar values than traditional cookies, while cookies elaborated with chia and poppy flours received the least positive evaluations.

Hinestroza-Córdoba et al. [16] evaluated the use of vacuum impregnation and high-pressure homogenization in order to obtain functional ingredients from lulo fruit (*Solanum quitoense* Lam.). Specifically, they studied the physicochemical and antioxidant properties of the lulo fruit and its juice. In their results, they found differences between the fruit and the juice and concluded that high-pressure homogenization increases the antiradical capacity of the juice and the diversity of polyphenols.

Zhang et al. [17] studied the willingness of consumers to pay for enhanced mandatory labeling of genetically modified soybean oil using a large sample of Chinese consumers. Their results show that Chinese consumers show positive attitudes towards traceability codes, reporting a willingness to pay of RMB 8.92 and for the labelling of allergen presences (RMB 6.57). In their conclusions, they state that policy strategies for enhanced mandatory labelling in genetically modified soybean oil will benefit consumers.

Brugarolas et al. [18] analyzed the behavior of consumers during the COVID-19 pandemic in order to create innovative strategies for the agri-food sector to cope with this new scenario. They found that 61% of consumers modified their buying behavior during lockdown, with increasing food stockpiling being the most common observed change. Regarding the degree of change in buying behavior, four different consumer segments were identified. The authors propose new strategies that companies could implement in order to deal with this new scenario, including the creation of a larger stock of non-perishable foods, increasing production capabilities, or boosting online sales.

Jiménez-Ortega et al. [19] evaluated the health-promoting properties of crocetin in order to promote its consideration as a healthy natural colorant. Specifically, they evaluated the ability of crocetin to reduce lipid accumulation during the differentiation of 3T3-L1 preadipocytes. In their results they state that 5 μM of crocetin decreased intracellular fat by 22.6% without affecting viability or lipid droplet generation. This result encourages the use of crocetin in dietary therapies intended to reverse adipose tissue accumulation in obesity cases.

Rabadán et al. [20] studied the effect of a melon cultivar (*Cucumis melo* L.) and an oil extraction method on the nutritional quality of melon seed oil. Nine different melon cultivars and two oil extraction methods (hydraulic and screw press) were evaluated. The results show that higher oil yields were obtained using the screw press. However, oils obtained with the hydraulic press resulted in higher quality oils. The obtained oils showed significant differences in their linoleic (51–69%) and oleic (15–34%) acids content. Vitamin E content also showed significant differences among cultivars. γ-tocopherol was the main isoform found in oils (range 99.81–456.73 mg/kg), followed by α- and δ-tocopherols. The principal-component analysis concluded that cultivars *Honew Dew* and *Blanco de Ribatejo* show the most promising characteristics to produce high-quality melon seed oils.

Roncero et al. [21] presented a review about almond kernel composition, making specific reference to the non-lipid components that traditionally have received less attention in the literature. They conclude that almonds are rich in proteins (8–35%), including important concentrations of the globulin-albumin fraction. The carbohydrate fraction (14–28%) is mainly compound by soluble sugars (such as sucrose) and starch. Regarding mineral elements, relevant concentrations of potassium and phosphorus were reported.

All of the above studies result in a wide multidisciplinary approach about the current state of the art in food innovation. The development of novel ingredients, foods, and innovative food production technologies, while simultaneously considering consumers’ perception of food innovation, will be crucial in order to produce healthier, safer, and more sustainable foods in the future.
